# Whole-head high-density diffuse optical tomography to map infant audio-visual responses to social and non-social stimuli

**DOI:** 10.1162/imag_a_00244

**Published:** 2024-09-11

**Authors:** Liam H. Collins-Jones, Louisa K. Gossé, Borja Blanco, Chiara Bulgarelli, Maheen Siddiqui, Ernesto E. Vidal-Rosas, Nida Duobaitė, Reuben W. Nixon-Hill, Greg Smith, James Skipper, Tim Sargent, Samuel Powell, Nicholas L. Everdell, Emily J.H. Jones, Robert J. Cooper

**Affiliations:** DOT-HUB, Department of Medical Physics and Biomedical Engineering, University College London, London, United Kingdom; Department of Medical Physics and Biomedical Engineering, University College London, London, United Kingdom; Department of Clinical Neurosciences, University of Cambridge, Cambridge, United Kingdom; Centre for Brain and Cognitive Development, Birkbeck University of London, London, United Kingdom; Department of Psychology, University of Cambridge, Cambridge, United Kingdom; Gowerlabs Ltd., London, United Kingdom

**Keywords:** high-density diffuse optical tomography, functional near-infrared spectroscopy, infant neuroimaging, optical neuroimaging, infant cognitive development

## Abstract

Infancy is a time of rapid brain development. High-density diffuse optical tomography (HD-DOT) is an optical neuroimaging method that maps changes in cortical haemoglobin concentration, a marker of functional brain activation. Recent years have seen a huge advance in wearable hardware for HD-DOT, however previous headgear has only been capable of sampling specific areas of the cortex. In this work, we aimed to develop headgear capable of sampling across the whole infant scalp surface and to conduct a proof-of-concept demonstration of whole-head HD-DOT in infants aged 5 to 7 months. We developed a whole-head infant implementation of the high-density LUMO design developed by Gowerlabs Ltd. (UK). HD-DOT data were collected from a cohort of infants (N = 16) during the presentation of a screen-based paradigm assessing social processing. Using whole-head HD-DOT, we mapped activity across the entirety of the optically-accessible cortex which far exceeds coverage achieved by previous infant optical neuroimaging methods. We found activity in temporal regions which corroborates previous research. Further, we mapped activity in regions outside those typically sampled in infant research using social processing paradigms, finding activation in regions across the occipital, parietal, and frontal cortices as well as an apparent inverted response in sensorimotor regions. Following this proof-of-concept, we envisage that whole-head HD-DOT will be applied to map the interaction between different regions of the brain, opening new avenues to map activity in the awake infant brain to better understand the trajectory of typical and atypical neurodevelopment.

## 1 Introduction

The first thousand days of life, from conception to 2 years of age, is a critical period in the development of the human brain (Bornstein, 2014;Cusick & Georgieff, 2012;Mendez & Adair, 1999;Powell et al., 1995). It is during this time that the brain is at its most plastic, undergoing cortical specialisation to adapt to the infant’s environment (Johnson, 2011). Studying the infant brain during this period is therefore crucial to understanding the emergence of different brain functions and how the trajectory of brain development differs between typically and atypically developing infants.

Research using functional near-infrared spectroscopy (fNIRS) has been instrumental to improving our understanding of the social brain network development in infancy. fNIRS is an optical imaging technique whereby the head is interrogated with near-infrared light via an array of sources and detectors placed on the scalp (Lee et al., 2017;White & Culver, 2010). Key advantages of fNIRS include that it is silent, non-invasive, portable, and relatively tolerant of motion (Eggebrecht et al., 2014;Ferradal et al., 2016;Lee et al., 2017), making this technique particularly well-suited for studying the brain in awake infants.

A key region of interest in the social brain network has been the posterior superior temporal sulcus-temporoparietal junction. The past 15 years have seen the development of paradigms (e.g., seeLloyd-Fox et al., 2009,^2012^) for fNIRS studies to illicit reproducible responses to social and non-social stimuli in this region.

Diffuse optical tomography (DOT) is an advancement of fNIRS where image reconstruction methods are applied to produce a three-dimensional image, which typically maps changes in haemoglobin concentration in superficial brain tissues including the cerebral cortex (White & Culver, 2010). The ability to acquire channels at a range of source-detector separations is essential to DOT approaches, as is having multiple channels sample each volume of tissue in the field-of-view. In high-density diffuse optical tomography (HD-DOT), a dense array of sources and detectors is used to sample changes in optical intensity. This increased density of sampling enables HD-DOT to approach a spatial resolution comparable to that of fMRI (Eggebrecht et al., 2012). A continuous distribution of channels with separations ranging from 10 to 40 mm also enables improved isolation of haemodynamics from non-brain activity, improving the specificity of signals originating from the cerebral cortex (Funane et al., 2014).

Recent years have seen a notable advance in wearable HD-DOT methods, which has demonstrated the potential of HD-DOT in a multitude of different research contexts. In adults, wearable HD-DOT has been applied in the home to investigate repeated measures of resting-state functional connectivity (Uchitel et al., 2022) and to probe visual functional responses (Vidal-Rosas et al., 2021). In infants,Frijia et al. (2021)published the first implementation of the high-density modular LUMO system (Gowerlabs, UK) in infants, applying a high-density wearable array (totalling 36 sources and 48 detectors) to map the haemodynamic response to human-generated and non-human-generated sounds in infants aged 4 to 7 months. Since then, wearable HD-DOT has been applied to map functional connectivity during sleep in newborn infants in clinical intensive care (Uchitel et al., 2023), demonstrating the breadth of contexts in which wearable HD-DOT can be applied.

A key limitation of previous fNIRS and HD-DOT studies has been the relatively small field-of-view of the fNIRS instrumentation, limiting the ability to investigate how other cortical structures are involved, which would provide a more complete picture of brain function. This is particularly important in social processing, where auditory and visual cues—perceived at different cortical locations—are integrated.

In this work, we aimed to achieve the first ever whole-head HD-DOT measurements in infants to map cross-cortex measures of brain activation in a cohort aged 5 to 7 months. We applied an audio-visual paradigm, similar to those used in fNIRS research with infants in this age-range, to illicit responses to social and non-social stimuli. We predicted that we would replicate findings from previous infant fNIRS investigations of social processing, finding bilateral temporal activation to audio-visual stimuli. We further aimed to examine activity in brain regions outside those typically sampled in fNIRS studies investigating social brain development.

## 2 Methods

### 2.1 Participants

Twenty-four healthy, term-born infants aged between 5 and 7 months of age were recruited for the study. Data collection took place at the ToddlerLab, Centre for Brain and Cognitive Development (CBCD) at Birkbeck, University of London (https://cbcd.bbk.ac.uk/toddlerlab). Written, informed consent was obtained from a parent/guardian for each participant prior to their participation. Ethical approval for this study was granted by the Departmental Ethics Committee of the Department of Psychological Sciences at Birkbeck, University of London (approval reference number 2122017).

### 2.2 HD-DOT system and cap design

We developed a whole-head infant head cap to support the high-density LUMO design developed by Gowerlabs Ltd. (UK). The LUMO system consists of multiple independent hexagonal modules (or “tiles”), each containing three dual-wavelength LED sources (emitting light at 735 and 850 nm) and four photodiode detectors, which are clipped into “docks” that are embedded in a neoprene cap.

The head cap was designed specifically for this study to sample across as wide an area as possible across the scalp. We initially assessed how many tiles could be placed on the scalp using a 45 cm head circumference phantom model of the infant head. For the final design, the neoprene cap contained 33 docks, meaning that 33 tiles could be clipped into the cap, totalling 99 sources and 132 detectors when all docks were filled with a tile. Two caps were produced for two different ranges of head circumferences (42–44 cm and 44–46 cm). The total weight of each cap with 33 LUMO tiles in place was approximately 480 g. The completed whole-head HD-DOT headgear is shown inFigure 1a and b. Intra-tile measurements were obtained with source-detector separations of approximately 10 mm and 20 mm (seeFig. 1f). Initially, between-tile measurements were acquired for all possible source-detector separations within the array (totalling 13,068 dual-wavelength channels), with an acquisition rate of 4.17 Hz. Data were collected at this frame rate for the first 5 participants recruited to the study. A firmware upgrade released during this study enabled an alternative acquisition mode operating at 8.33 Hz. The increase in frame rate is achieved by discarding some of the longest source-detector separations (>60 mm) which are often too distant to obtain useful measurements. This reduced the total number of dual-wavelength channels recorded to 5,544.

**Fig. 1.**
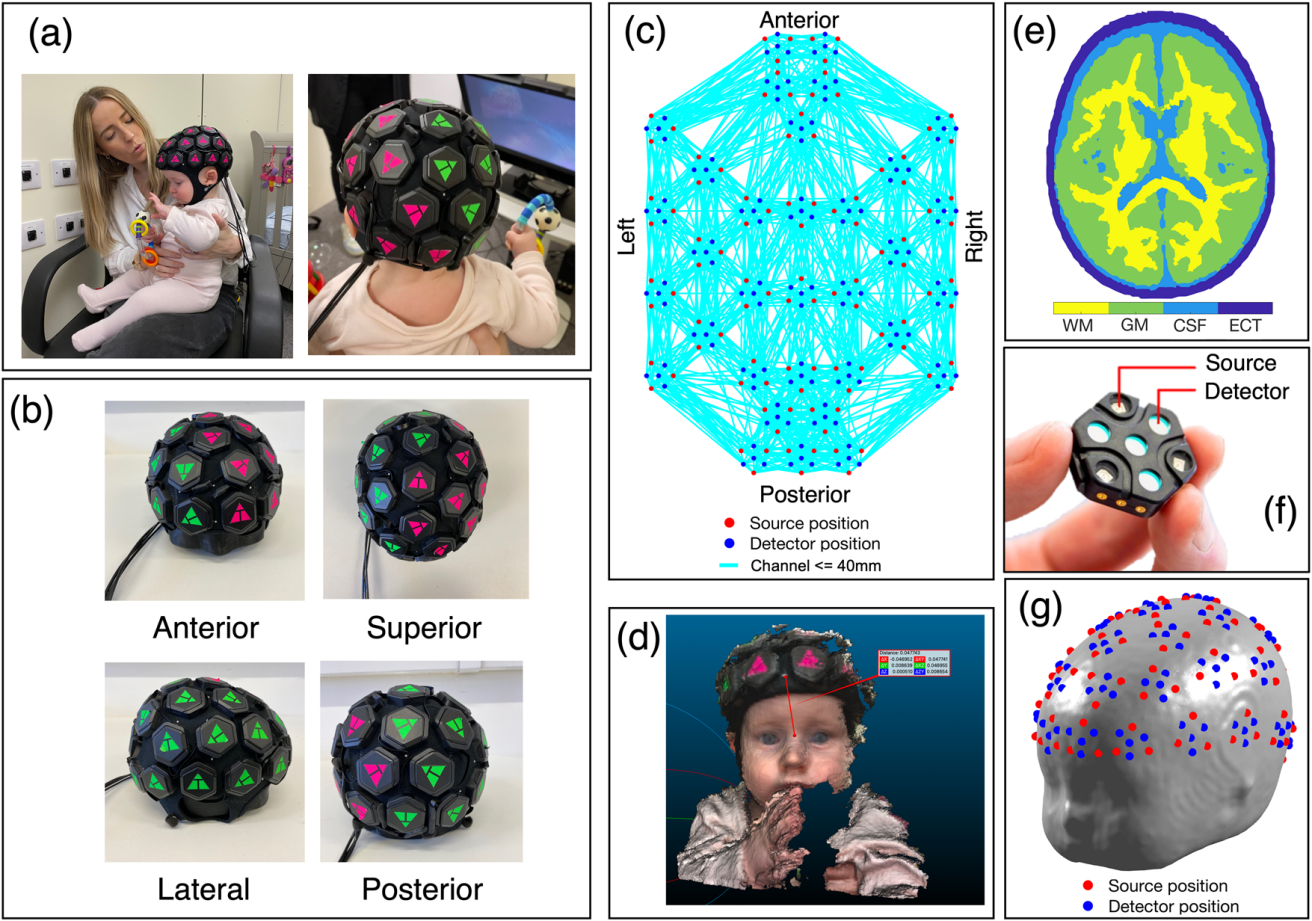
(a) 33-dock whole-head HD-DOT cap on a 7-month-old infant’s head. (b) Anterior, superior, lateral, and posterior views of the infant whole-head cap on a phantom. (c) Channel map for source-detector separations of 40 mm and below. (d) Anterior true-depth scan of an infant’s head, measuring the position of a point on the cap (intended to lie on the midline) relative to the nasion. (e) Axial slice of the tetrahedral volume mesh showing spatial distribution of white matter (WM), grey matter (GM), cerebrospinal fluid (CSF), and extra-cerebral tissue (ECT). (f) LUMO tile, consisting of four photodiode detectors and three dual-wavelength sources. Source-detector distances within each tile are 10 mm and 20 mm. (g) Source and detector positions registered to the infant scalp surface for an example individual.

### 2.3 Cap placement

Each infant participant’s head circumference was measured around the crown to inform cap selection. The distance between the left (Al) and right (Ar) pre-auricular points via the apex of the head (Cz) was also measured, as was the distance between the nasion (Nz) and the inion (Iz) via Cz. For infants with a head circumference of 44 cm or below, cap placement on the infant head was attempted with the 42–44 cm cap. For infants with a head circumference above 44 cm (or who had issues during cap fitting with the smaller cap), the 44–46 cm cap was placed on the infant head. The cap was first fitted to align the ears through the ear gaps to ensure the cap was level, and a white dot at the front of the cap was in line with the nasion. The cap was secured on the infant head with Velcro™ straps under the participant’s chin.

### 2.4 Head modelling

A four-layer tetrahedral mesh model of the infant head was used in this work (seeFig. 1e), which we developed and employed in a previous HD-DOT demonstration in infants (Frijia et al., 2021). This model was produced using averaged structural MRI data from a cohort of 6-month-old infants, and maps the spatial distribution of grey matter, white matter, cerebrospinal fluid, and extra-cerebral tissue within the head. The data to produce this head model were obtained from the Neurodevelopmental MRI Database (Almli et al., 2007;Richards et al., 2016;Sanchez et al., 2012), originally deriving from the NIHPD Objective-2 and MCBI-USC databases. A tetrahedral volume mesh and a cortical surface mesh were produced using iso2mesh (Fang & Boas, 2009). A scalp surface mesh was also produced, on which the positions of cranial landmarks (Nz, Iz, Ar, Al, and Cz) were defined.

For each participant, the mesh model was scaled iteratively to the participant’s head circumference, Nz-Cz-Iz and Ar-Cz-Al measurements, similar to the process described inCollins-Jones et al. (2021). The head model was reoriented such that a line from the inion (denoted as the origin) to the nasion defined the y-axis, and the head model was rotated around the y-axis such that the pre-auricular points had the same z-coordinate value. Initially, a scale factor was computed by dividing the participant’s head circumference by the mesh model’s circumference, then multiplying the node coordinates by this scale factor. The head circumference, Nz-Cz-Iz distance, and Ar-Cz-Al distance were then measured from the scaled mesh model; the measurement with the greatest discrepancy relative to the participant measured value was then divided by the participant measured value to compute a new scale factor. If the Nz-Cz-Iz measurement had the greatest discrepancy, the y- and z-coordinates were multiplied by the scale factor; if the Ar-Cz-Al had the greatest discrepancy, the x- and z-coordinates were multiplied by the scale factor. This process was repeated until all measurements were within 5 mm of the participant-measured values.

### 2.5 Optode registration and forward modelling

After this iterative scaling process, source-detector positions were registered to the head model. A template set of source-detector positions and cranial landmarks positions were determined on a phantom for each cap using a Polhemus PATRIOT™ digitiser system (Polhemus, USA). The template positions of the tile centres were then transformed to the space of the scaled mesh model using an affine transform between the phantom’s and scaled mesh model’s cranial landmarks.

To gain participant-specific cap placement measures, depth-resolved scans of the head were taken using the TrueDepth camera functionality of an iPhone 11 (Apple, Inc., USA) and the app Scandy Pro (Scandy LLC, USA), which together allowed scaled, three-dimensional point clouds to be acquired. Snapshots of approximately 1–2 s were acquired of the anterior of the head, which included the nose, eyes, and bottom of the cap. A white dot on the anterior of the cap denoted the middle of the cap (seeFig. 1d), and the cap placement was intended to ensure that this dot lay directly above the nasion. Using the CloudCompare software package (www.danielgm.net/cc), the position of this dot relative to the nasion was determined for each participant from their anterior snapshot point cloud.

The offset between the template position and measured position of the white dot was used to compute rotations around the x- and z-axes. These rotations were applied to the module centres on the scaled mesh model, and the nearest node on the mesh surface was then found for each module centre. Assuming a fixed within-tile distance, the source and detector positions for each tile were then registered to the scalp surface relative to the tile centre positions (seeFig. 1g).

Depth-resolved images of array placement were not acquired from one participant in the final sample, and so the template positioning of the array on a 6-month head phantom was used in place of participant-specific measurements.

### 2.6 Experimental paradigm

Whole-head HD-DOT data were collected from infants during the presentation of an audio-visual screen-based paradigm. During presentation of the paradigm, infants were seated on a parent’s lap, approximately 70 cm from a screen. Paradigm presentation was coded using Psychtoolbox in MATLAB (Mathworks, USA) and controlled by a researcher from a MacBook Pro (Apple, Inc., USA) out of the infant’s view. A webcam was used to record the infant’s gaze towards the screen.

The paradigm included two experimental conditions—a*social*condition and a*non-social*condition—as well as a baseline condition (seeFig. 2). The social condition consisted of full-colour video clips of actors performing nursery rhymes, and the non-social condition consisted of dynamic videos of moving mechanical toys. A matched audio component accompanied a visual component for both conditions. This same paradigm has been used previously in infant fNIRS (Siddiqui et al., 2022) and EEG (Jones et al., 2015) studies. Both experimental conditions were presented for a varying duration between 9 and 12 s. The baseline condition consisted of still images of different modes of transport (such as cars and helicopters), alternating every 1–3 s, which were presented for a total of 12–14.5 s. The baseline and experimental conditions were alternated, and were presented until the parent signalled to stop or a researcher qualitatively determined that the infant became visibly agitated and appeared unlikely to calm down. Up to 9 trials of each experimental condition were presented. The paradigm, if presented in its entirety, had a duration of approximately 7 min. During the study, and consistent with previous applications of the same paradigm in other studies, the infant’s parent/guardian was instructed not to interact with the infant. To re-direct the infant’s attention towards the screen if they became distracted, an alerting sound was played. The alerting sounds were used for two of the participants in the final sample immediately following the presentation of an experimental trial. Data from these trials were removed before analysis.

**Fig. 2.**
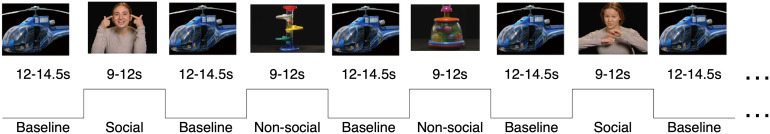
Order of stimulus presentation in the social/non-social paradigm. Up to 9 trials of each experimental condition (social and non-social) were presented, interleaved between the baseline condition trials. The paradigm in its entirety lasted approximately 7 min.

### 2.7 Data pre-processing

Infant gaze towards the screen was coded manually offline to quantify the total screen-directed looking time during each trial. The infant was required to look at the screen for a minimum of 60% of the experimental trial period for the trial to be considered valid, and a minimum of three valid trials for each experimental condition were required to include the infant in the final sample (Lloyd-Fox et al., 2014).

Data pre-processing steps were completed using the Homer2 toolbox ( https://homer-fnirs.org/ ; Huppert et al., 2009 ). Within each participant, three criteria were used to define a good channel based on a segment of data where no motion artifacts were apparent on visual inspection:

- Coefficient-of-variation (defined as the standard deviation of intensity divided by the mean intensity) below 8%
- Intensity threshold greater than 5 x 10^-5^V, based on manufacturer’s recommendation and as used byUchitel et al. (2022)
- Source-detector separation below 60 mm.

Channels failing to meet these criteria were excluded. Optical intensity data were converted to changes in optical density (ΔOD) by dividing each channel’s intensity time-course by its mean, then taking its natural log, and multiplying by -1 (this was completed using the*hmrIntensity2OD*function).

To identify channels with motion artifacts, the*hmrMotionArtifactByChannel*function was used. If, within a pre-defined time-window (*tMotion*, set to 1.0 s) for a given channel, the change in ΔOD signal was greater than an amplitude threshold (*AMPthresh*, set to 0.5) or if the standard deviation surpassed a threshold (*STDEVthresh*, set to 15), then the data points during that time period (±*tMask*, set to 1.0 s) were marked as motion artifact.

We chose to implement spline interpolation and wavelet transformation (as well as the input values) following the work published byDi Lorenzo et al. (2019).

Segments of data flagged as motion artifact by*hmrMotionArtifactByChannel*were then corrected using cubic spline interpolation, a method first demonstrated byScholkmann et al. (2010). This interpolation was subtracted from the original ΔOD signal and then baseline corrected to ensure a continuous data time-course for data preceding and following the motion artifact. For this step, we used*hmrMotionCorrectSpline*and assigned a value of 0.99 for the parameter used in the spline interpolation.

A wavelet transformation was computed, which decomposed each channel’s ΔOD time-course into a series of Gaussian wavelet bases (Molavi & Dumont, 2012). Bases associated with neural haemodynamics will be distributed around zero, but bases representing motion artifacts can be identified as outliers of the Gaussian distribution lying outside the interval [first quartile –a*iqr, third quartile +a*iqr]. Here, iqr is defined as the interquartile range and*a*is a user-defined parameter (in this work, set to 0.8). Coefficients identified as outliers are then set to zero before the signal is recomposed of the modified coefficients to obtain a version of the signal less influenced by motion artifacts. This was computed using the*hmrMotionCorrectWavelet*function.

For each experimental condition, ΔOD data for each channel were block-averaged from 2 s pre-stimulus to 20 s post-stimulus onset and then downsampled from the original acquisition frequency (either 4.17 Hz or 8.33 Hz) to 4 Hz using linear interpolation. This was completed using the*hmrBlockAvg*function.

We block-averaged ΔOD data across channels in each participant before reconstructing. From a practical and efficiency perspective, the purpose of using this approach was to demonstrate a method that minimised computational time and requirements for computational power and memory storage. This is important in the context of whole-head HD-DOT where data are acquired from a substantially higher number of channels than conventional fNIRS and HD-DOT approaches, and this block-averaging approach exhibits the feasibility of whole-head infant HD-DOT to be applied by researchers without requiring extensive computational facilities.

### 2.8 Image reconstruction

A zeroth-order Tikhonov regularised reconstruction was performed using a regularisation hyperparameter of 0.01. Block-averaged ΔOD data (downsampled to 4 Hz) for both experimental conditions were reconstructed to produce images mapping the changes in oxy-haemoglobin (HbO) and deoxy-haemoglobin (HbR) concentrations relative to a 2 s pre-stimulus baseline period.

All images were reconstructed in the space of the tetrahedral volume mesh, then mapped to the cortical surface by taking a mean of concentration change values within a 3 mm radius of each cortical surface node. Code used to pre-process and reconstruct the HD-DOT data is freely available as part of the DOT-HUB toolbox atwww.github.com/DOT-HUB.

### 2.9 Coverage threshold

A coverage threshold was defined to determine whether the whole-head array can be considered sensitive to a particular node. Based on a metric defined byBrigadoi et al. (2018), the array was considered to be sensitive to a node when a change in the absorption coefficient ($\Delta \mu_{a}$) of 10% in a 1 cm^3^block of tissue ($act_{vol}$) would lead to a >1% change ($p_{thresh}$) in measured intensity. A coverage threshold was computed for each individual using the warped mesh’s mean Voronoi volume (V) using the following equation:

$$coverage\;threshold=\;\frac{\text{log}\left(\frac{100+p_{thresh}}{100}\right)}{\frac{act_{vol}}{\hat{V}}*\Delta \mu_{a}}$$

Each individual’s Jacobian was thresholded by this value to produce a binarised sensitivity mask. These masks were then summed on a node-wise basis across individuals. In group-level images, data were included only from nodes that were covered in at least 75% of participants in the final sample.

### 2.10 Statistical analyses

We produced t-statistic maps of the block-averaged group-level response to each experimental condition during a time window that represented the peak time of response. A time window of 11–15 s post-stimulus onset was used to capture the peak of the haemodynamic response in the image time-course. This choice of window was informed by visually inspecting the block-averaged time-courses in different regions across the cortex and is consistent with prior applications of this paradigm (Frijia et al., 2021;Lloyd-Fox et al., 2017).

For each experimental condition, chromophore concentration change values (for both HbO and HbR) across participants within the response window were concatenated to yield a single vector for each node in the cortical surface mesh. For each condition and chromophore, response vectors for each node were then tested using a one-sample t-test. Only nodes covered in ≥75% of participants in the final sample were tested. Concentration change values during this window were also compared between the two experimental conditions using a paired-sample t-test.

## 3 Results

### 3.1 Participants

Of the N = 24 participants recruited for the study, a total of 8 infants were excluded for the following criteria:

- Not enough trials collected due to experimenter error (N = 1 excluded)
- Not enough valid trials passing looking time threshold (N = 1 excluded)
- Disconnection between tile and prototype cap during data collection leading to software error (N = 3 excluded)
- Cap not fitting securely (N = 3 excluded)

In total, our final sample size included N = 16 infants aged 5 to 7 months (11 female, 5 male). Demographic information for the cohort is displayed inTable 1. The attrition rate of this study is typical of what is seen in infant fNIRS research (Baek et al., 2023).

**Table 1.** Demographic information for infants in final sample.

	Mean	SD	Range
Age (days)	188.1	27.7	153-237
Head circumference (cm)	43.3	1.3	41-45
Left to right pre-auricular points via Cz (cm)	27.7	1.7	24-31
Nasion to inion via Cz (cm)	28.0	1.0	27-30

### 3.2 Looking time measures

One infant was excluded following looking-time analysis as they did not have enough valid trials (i.e., three per condition). Of the 16 infants included in the final sample, the median number of trials with looking times above the 60% threshold was 8 (range 4–9) for the social condition and 7 (range 3–9) for the non-social condition. The median looking time for accepted trials for the social condition was 10.2 s (range 6.8–12.0 s) and for the non-social condition was 9.7 s (range 6.3–12.0 s).

### 3.3 Metrics of system performance

#### 3.3.1 Signal quality and channel count

**Fig. 3.**
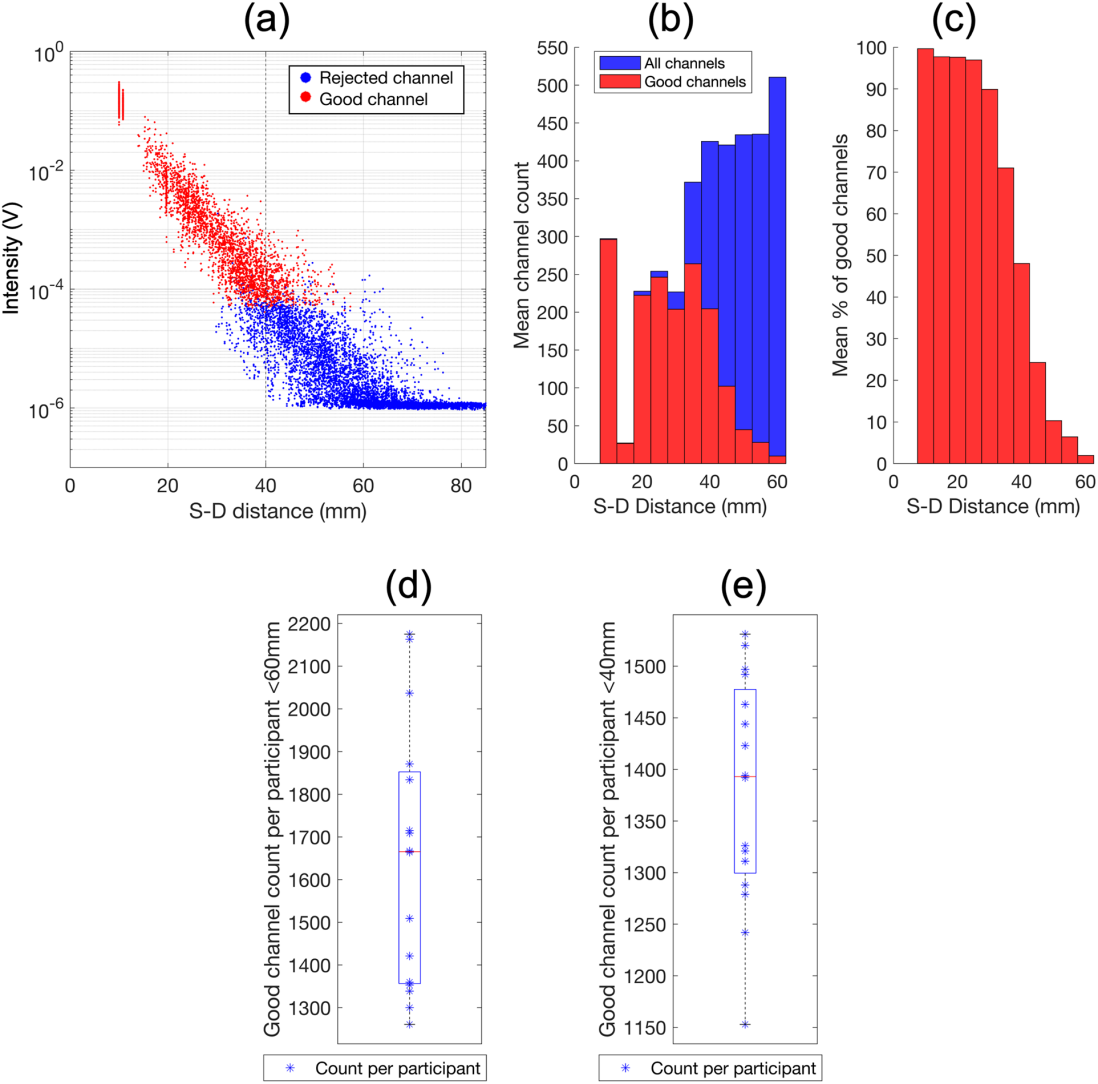
Performance metrics of the infant whole-head HD-DOT system. Mean intensity over the time-course for each source-detector pair at each wavelength as a function of source-detector separation for an example participant is plotted on a logarithmic scale in (a); the dotted line is plotted at distance 40 mm which is the distance threshold for channels used in image reconstruction. Histogram of mean channel count across all participants as a function of source-detector separation is shown in (b). Histogram of the mean percentage of good channels (i.e., channels passing the coefficient-of-variation threshold) as a function of source-detector separation is shown in (c). Number of good channels <60 mm per participant plotted in (d), and number of good channels <40 mm per participant plotted in (e).

Figure 3adisplays the mean detected intensity across the time-course for all channels at both wavelengths as a function of source-detector separation for an example participant. These intensity values follow an expected log-linear decay. Channels passing the coefficient-of-variation threshold (without showing saturation) are shown in red, while the remaining (excluded) channels are shown in blue.Supplementary Figure S1shows the same data for all participants.

Figure 3bshows a histogram of the mean channel count of dual-wavelength channels (i.e., fNIRS channels) surviving the coefficient-of-variation threshold.Figure 3cdisplays the mean percentage of good channels recovered as a function of source-detector separation. The mean percentage decreases as source-detector separation increases, however 90% of channels are being recovered in the bin centred at 30 mm. The number of good channels below 60 mm obtained for each participant is plotted inFigure 3d, while the same is shown inFigure 3efor good channels below 40 mm. The mean number of good channels obtained per participant across the group was 1,380 (range 1,153–1,531) for channels below 40 mm and 1,649 (range 1,261–2,175) for channels below 60 mm.

#### 3.3.2 Coverage

For each participant, the coverage threshold (defined inSection 2.9“Coverage threshold”) was applied to compute a binarised sensitivity mask for each participant using good channels at different source-detector length intervals: 10–20 mm (the shortest channels, including all intra-tile channels and the shortest between-tile channels), 20–30 mm, 30–40 mm, 40–50 mm, and 50–60 mm. To quantify the consistency of coverage across participants, we summed the binary sensitivity masks across participants for each source-detector interval, shown inFigure 4.

**Fig. 4.**
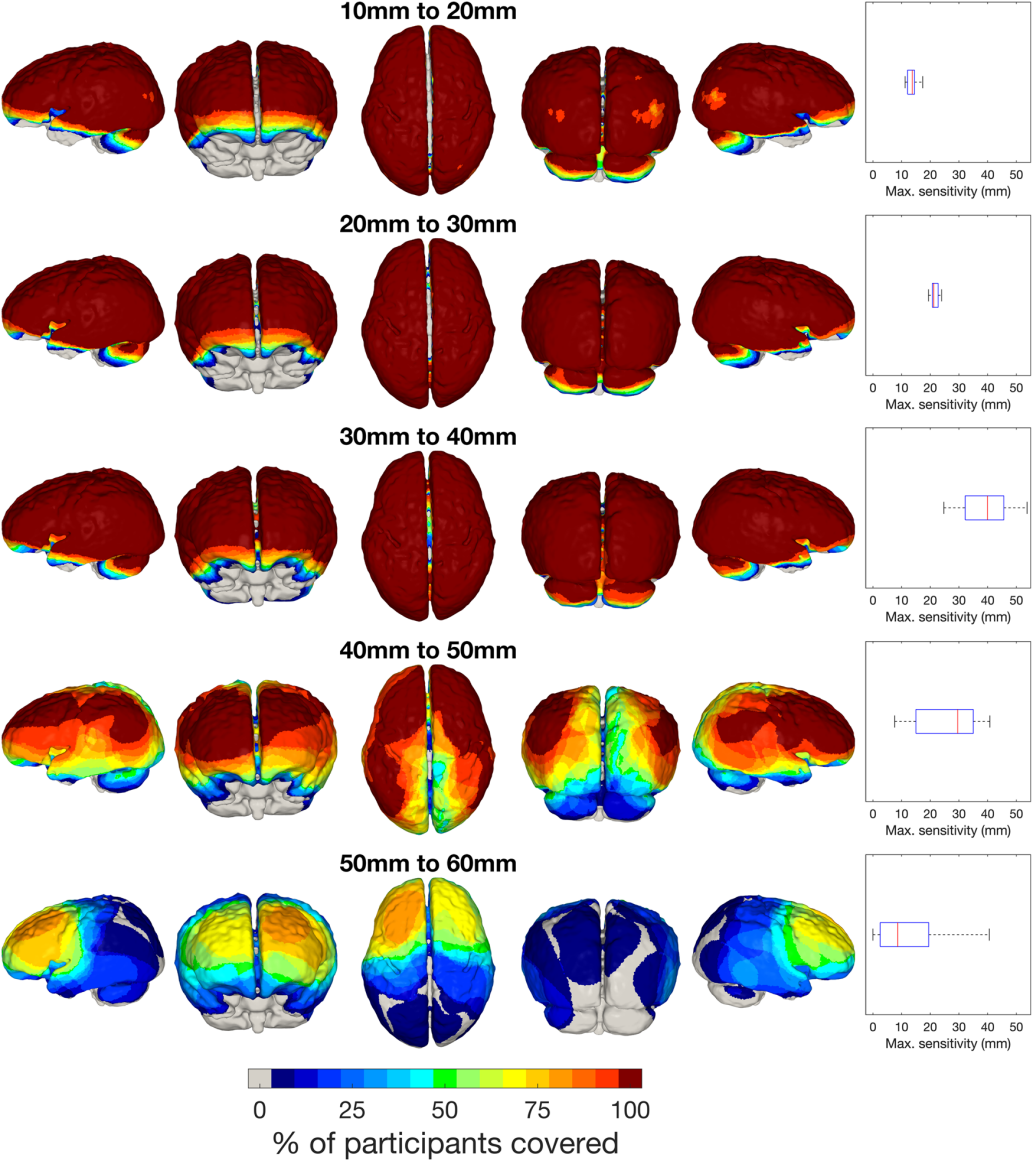
Sum of binarised sensitivity masks across participants for channels at different source-detector separation intervals. The far-right column of the figure plots the distribution of the maximum cortical sensitivity values across participants for each source-detector separation interval.

For the 10–20 mm channels, we see widespread coverage of the cortex except for a region in the lateral occipital cortex which appears to coincide with the largest gap between tiles in the cap. Given the infant scalp-to-brain distance, a reasonable level of coverage at 20 mm is expected in infants of this age-range. For the 20–30 mm interval, we see widespread coverage of the cortex across participants; the same is true for the 30–40 mm interval. For the 40–50 mm interval, we see regions with consistent coverage across participants in frontal and inferior parietal areas, but in general the consistency of coverage drops off significantly from anterior to posterior regions of the cortex. Above 50 mm, a similar pattern is seen but with much lower consistency across participants. These patterns in cortical coverage are reflected inSupplementary Figure S2which displays the spatial distribution of good channels across participants.

Though cortical coverage is similar for the lower three intervals in terms of nodes passing the sensitivity threshold, the maximum sensitivity value in the cortex increases for each interval up to 30–40 mm (see far-right column inFig. 4). For the source-detector separation intervals greater than 40 mm, the peak cortical sensitivity value across participants is far more variable and shows a tendency to decrease, owing to the marked drop in good channels (see the 40–50 mm and 50–60 mm good channel map inSupplementary Figure S2).

To maintain consistency in the face of large variability in coverage above 40 mm, we decided to restrict image reconstruction to channels up to a source-detector length of 40 mm.Figure 4displays the sum of binarised sensitivity masks across individuals for good channels up to 40 mm across participants. In each participant, widespread coverage is seen in the temporal, frontal, parietal, and occipital lobes. There is variable coverage of the inferior-most regions of the temporal lobe and the anterior-most regions of the frontal lobe. There is also variable coverage of the cerebellum, though cerebellar coverage was not intended.

### 3.4 Block-averaged functional images

#### 3.4.1 Statistical mapping

Figure 6displays t-statistic maps of the response to the social and non-social conditions. A positive t-statistic value indicates that the mean changes in chromophore concentration during the 11–15 s post-stimulus window are significantly higher than a distribution with a mean of zero, though it does not necessarily require that these changes are associated with a canonical haemodynamic response. A highly conservative Bonferroni correction was performed based on the number of cortical nodes covered in ≥75% of participants in the final sample, similar to the approach taken byFrijia et al. (2021). A concurrent increase in HbO and decrease in HbR concentrations is a marker of functional brain activation. Maps of haemoglobin concentration changes can be found inSupplementary Figure S3.

In the following text, we will refer to arrows and circles inFigure 6. In response to both conditions, the mean of the HbO response was significantly increased in the temporal, occipital, parietal, and frontal lobes. The largest effect size was seen overlying the right superior temporal suclus (arrow A) and the left temporoparietal junction (arrow B) for the social condition where concurrent increases in HbO and decreases in HbR concentrations are seen. Significant changes for the non-social condition appeared across a greater area of the temporal lobe, particularly in the right hemisphere where a focus appeared anteriorly (arrow C). Changes in HbR concentration in the temporal lobes appear to mirror the pattern for HbO.

In the posterior occipital lobe, increases in HbO concentration can also be seen for both experimental conditions. Significant HbO changes appear more spatially widespread (arrow D) in the non-social condition. Concurrent decreases in HbR concentration can be seen in medial occipital areas in response to the non-social condition but not for the social condition.

In the right inferior frontal gyrus, significant increases in HbO can be seen in response to both conditions (arrows E and F), where significant changes appear over a larger area in response to the non-social condition. Significant decreases in the left inferior frontal gyrus are seen in response to both conditions.

In the medial frontal gyrus, an area bilaterally with a significant increase in HbO concentration is seen in response to the social condition (arrow G) which is not present in the non-social condition. This is also true for changes in HbR concentration in response to the social condition, though these changes occur marginally further anteriorly.

An apparent inverted response is present in the sensorimotor regions superiorly (pre- and post-central gyri), where a decrease in HbO concentration and an increase in HbR concentration occur. This is most prominently seen in the significant HbO concentration decreases in both experimental conditions (present over a larger area in the social condition, see circles labelled H), while significant increases in HbR concentration are present over a much smaller area.

Figure 7compares the response to the non-social condition (in an 11–15 s activation window) against the response to the social condition using a paired t-test, also corrected using a highly conservative Bonferroni approach based on the number of cortical nodes covered in ≥75% of participants in the final sample. In the figure, red colours indicate that the magnitude of the increase in HbO (or magnitude of decrease in HbR concentration) is greater in the non-social condition, while blue colours indicate that the magnitude of the increase in HbO (or magnitude of decrease in HbR concentration) is greater in the social condition. Mean increases in HbO concentration are significantly higher for the non-social condition across a wide area of the cortex, including areas in the frontal, occipital, and inferior temporal cortices (denoted by the red-coded t-statistic values). Changes in HbO concentration are significantly higher for the social condition (denoted by the blue-coded t-statistic values, arrow A) in the right posterior superior temporal sulcus.

#### 3.4.2 Peak changes and temporal dynamics

Figure 8displays group-level changes in HbO concentration in response to the social condition in an 11–15 s time window post-stimulus onset. In the group-level image, we found the cortical surface node with the greatest change in HbO concentration in three regions in each hemisphere: the temporal lobe, the inferior frontal gyrus, and the occipital lobe. These nodes were used to define a seed region which encompassed all cortical surface nodes within a 5 mm radius. Across nodes in each seed region, mean time-courses (for both HbO and HbR changes) were computed in each participant from their block-averaged reconstructed images, and these seed region time-courses were then averaged across participants; this is shown inFigure 8(the shaded area in the figure indicates the standard error of the mean computed across participants). The same was performed for the pre-central gyrus with the aim of capturing the apparent inverted haemodynamic response. In this region, the peak decrease in HbO concentration was used to define the node at the seed centre.

The resulting seed regions are shown inFigure 8for the social condition, underneath which are the mean response time-courses for the seed regions. A canonical haemodynamic response is seen in the temporal seeds from both hemispheres, showing an increase in HbO concentration (reaching its peak at approximately 13 s in the left hemisphere and 12 s in the right) and a concurrent decrease in HbR concentration. For the occipital seeds, increases in HbO concentration can be seen (reaching a peak at approximately 12–13 s), though there is no clear decrease in HbR concentration. More details on the apparent inverted response can be seen in the pre-central gyrus seed: an increase in HbR concentration and an even greater decrease in HbO concentration are seen, reaching a peak decrease at approximately 10–11 s which is just prior to the window chosen to represent the peak functional response. In the inferior frontal gyri bilaterally, there is no clear functional response.

The time-courses for the corresponding seed regions in response to the non-social condition are shown inFigure 9. Like the social condition, a canonical haemodynamic response is seen in the temporal seed of both hemispheres. Increases in HbO concentration can be seen in the occipital lobe which, like the social condition, do not show clear decreases in HbR concentration. There is evidence of a response in the inferior frontal gyrus, where an increased HbO concentration and a decreased HbR concentration are seen during the 11–15 s window. The changes in HbO and HbR concentrations seen in the motor seeds are of a lesser magnitude than those seen in the results for the social condition.

To ascertain more about the inverted sensorimotor responses in the experimental conditions, the same block-averaging analysis was repeated using the onset of the baseline condition (seeSupplementary Figure S5). Here, an increase in HbO concentration with a concurrent decrease in HbR concentration is seen in the motor seed, suggesting that motor activation is occurring in the baseline period, potentially due to movement as the infant becomes fussy with less stimulation compared to the experimental conditions.

## 4 Discussion

To our knowledge, we have conducted the first ever study of infant brain function using whole-head HD-DOT. We have mapped infant cortical responses to audio-visual social and non-social stimuli, and our results demonstrate that it is now possible to map cortex-wide activity in the awake infant brain outside the conventional scanning environment.

### 4.1 System performance

The data inFigure 3show that a high number of channels are being recovered. As shown inFigure 3c, ahigh percentage of channels were surviving the data quality checks (i.e., below coefficient-of-variation threshold, exceeding intensity threshold and source-detector separation <60 mm), with 90% of channels in the 27.5–32.5 mm bin passing these criteria. To place these distances in context, infant fNIRS arrays typically sample channels with a 20–25 mm source-detector separation. In all infants, despite the variability in channel count across participants (seeFig. 3d, e), wide coverage of the cortex was maintained as assessed by an objective metric of array sensitivity, even when restricting image reconstruction to channels <40 mm (as shown inFig. 5).

**Fig. 5.**
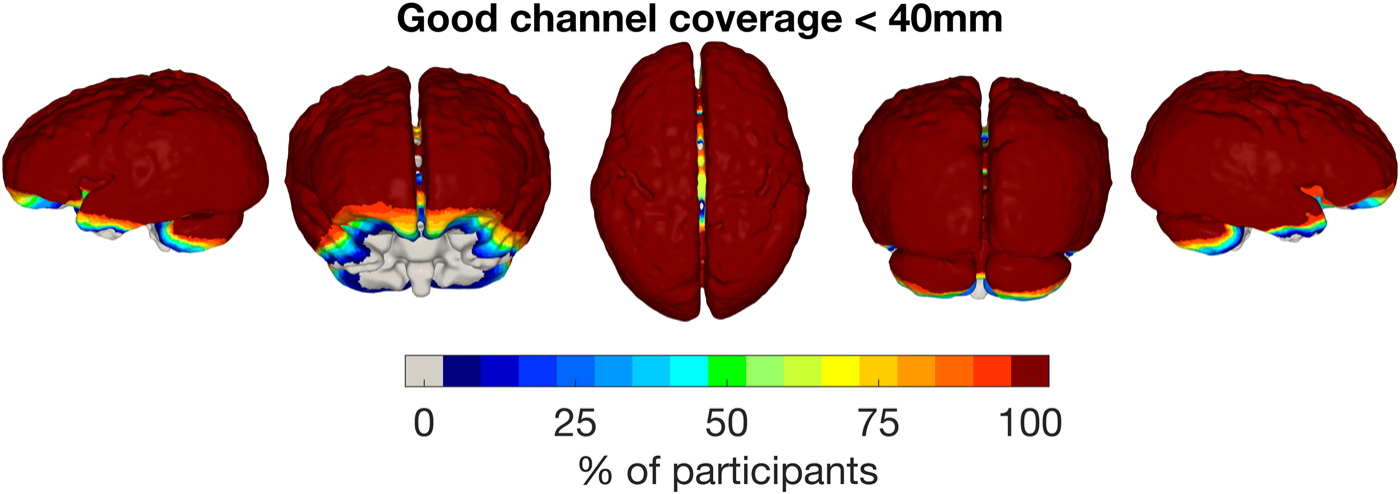
Array coverage of the cortex, showing the sum of binarised sensitivity masks across participants for channels <40 mm.

Despite these positive signs, the percentages of good channels obtained in this study were slightly worse than those observed byFrijia et al. (2021). This is not surprising given the limited field-of-view of the prior array provided more space for hair to be displaced into, while the whole-head cap used in this study had to achieve good coupling across the whole surface of the scalp (seeSection 4.3for further discussion on hair). The whole-head cap was secured with a Velcro^TM^chin strap, which was the only way to tighten the cap. The cap produced byFrijia et al. (2021)had other Velcro^TM^straps spanning between hemispheres to tighten the cap; however, in the present work, such straps were not included in order to maximise space for tiles. This lack of tightening mechanism likely led to worse contact between some of the tiles and the scalp, which would explain the decreased rate of channel retention. This was the first implementation of the whole-head cap in infants, and so a further solution may be to devise an improved protocol to optimally fit the cap to the infant head, or to include tightening straps in future designs.

In total, 24 participants were recruited and 8 were excluded, with only one participant excluded for not attending enough trials. This low level of participant exclusion, which is lower than the mean exclusion rate in infant fNIRS studies (Baek et al., 2023), demonstrates that the cap is well tolerated by infants. Because of the high channel retention rate, and the high sampling density provided by this technology, no participant was rejected because of intrinsically poor data quality.

### 4.2 Activation maps

In this work, we collected whole-head HD-DOT from infants during the presentation of audio-visual social and non-social stimuli. This paradigm and similar have been widely applied in fNIRS studies to investigate infant brain responses in a range of research contexts, such as in rural field-based studies (Lloyd-Fox et al., 2014); low-resource urban settings (Pirazzoli et al., 2022); longitudinal studies (Lloyd-Fox et al., 2017); and investigations of cerebral metabolism (Siddiqui et al., 2022). In our work, we have replicated previous findings on infant temporal lobe activity in this age-range as well as shedding light on activity outside areas typically sampled during these paradigms.

#### 4.2.1 Replication of previous findings in temporal regions

The block-average analysis demonstrated bilateral temporal activation in response to both conditions. This is consistent withLloyd-Fox et al. (2012),Blanco et al. (2023), andFrijia et al. (2021), who all found a bilateral response to voice and non-voice sounds in infants in a similar age-range.

**Fig. 6.**
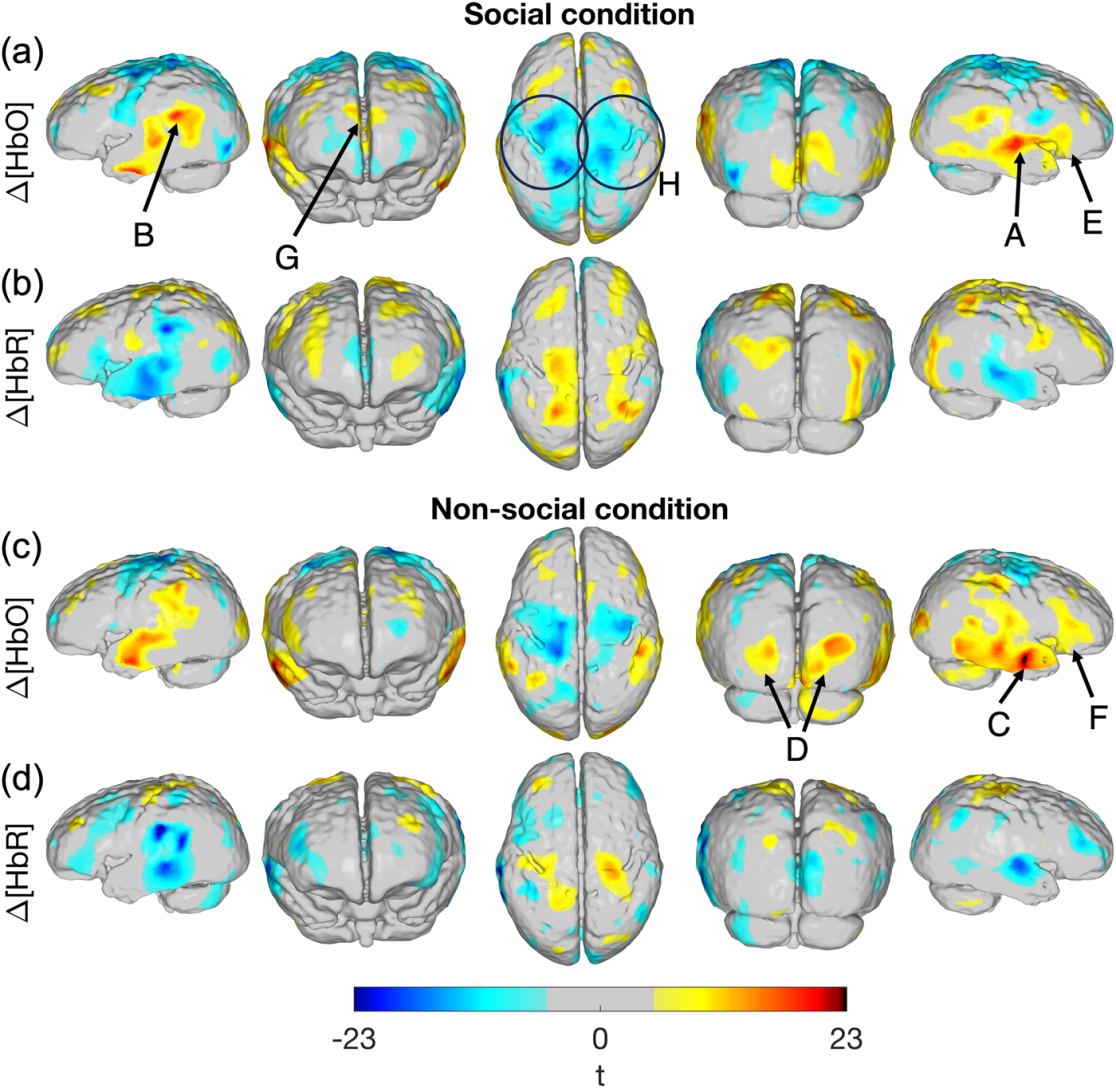
t-statistic maps of the HbO (a, c) and HbR (b, d) concentration changes during an 11–15 s post-stimulus window. Results are shown for the social condition (a, b) and the non-social condition (c, d). Changes shown for significance threshold*p*< 0.05 (Bonferroni corrected). Image reconstruction performed with data from good channels <40 mm. Description of arrows and circles: temporal activity overlying the posterior superior temporal sulcus (A), temporoparietal junction (B), and anterior temporal lobe (C); more widespread occipital activity in non-social condition (D); right inferior frontal gyrus activity (E and F); activity in medial frontal gyrus in social condition (G); inverted response in sensorimotor area (H).Supplementary Figure S4displays the same images but without the annotating arrows and circles.

Grossmann et al. (2010)found the posterior temporal area in infants to be voice-selective and found a right-lateralised response to voice, whileBlasi et al. (2011)found a right-lateralised temporal response to voice versus non-voice in infants. This is consistent with our results, where we found a larger response (see arrow A,Fig. 6) in the right posterior superior temporal sulcus (vs. left) for the social condition.

Lloyd-Fox et al. (2017)found a significantly greater HbO response in channels overlying the posterior superior temporal sulcus-temporoparietal region (pSTS-TPJ) in infants aged 4 to 8 months in response to voice versus non-voice.Figures 8and9indicate that the magnitude of the temporal concentration changes appears to be larger for social condition versus non-social condition, particularly in the right hemisphere; this is supported inFigure 7(see arrow A) where a small area overlying the right posterior superior temporal sulcus shows a significantly larger HbO response to the social versus non-social condition. Consistent with this, fMRI work in awake infants has found a preference to faces in the superior temporal sulcus (Deen et al., 2017).

**Fig. 7.**
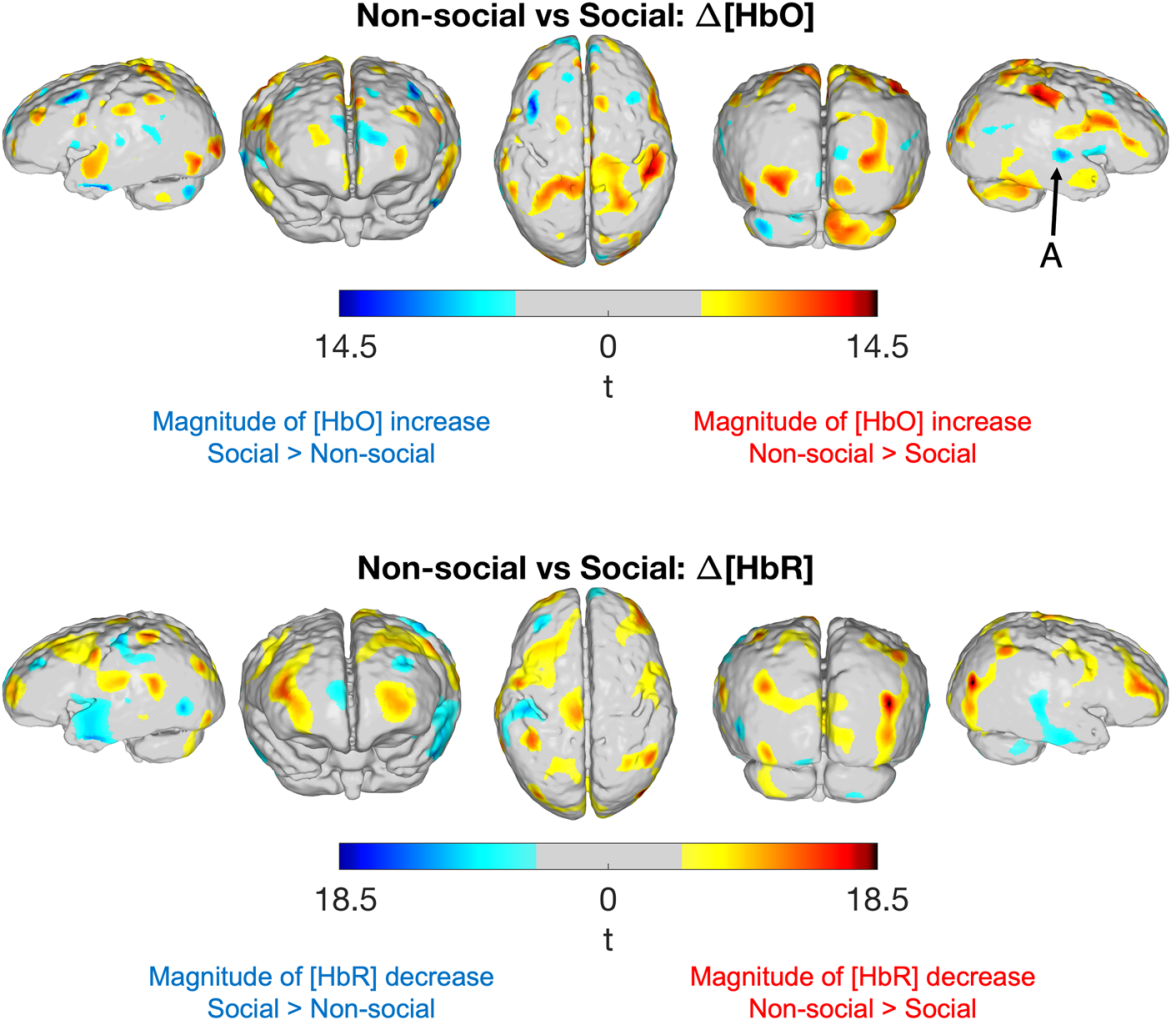
t-statistic maps comparing the mean HbO and HbR responses between experimental conditions in an 11–15 s window post-stimulus onset. Red colour-coded t-statistic values indicate that the that the magnitude of the increase in HbO (or magnitude of decrease in HbR concentration) is greater in the non-social condition, while blue colour-coded t-statistic values indicate that the magnitude of the increase in HbO (or magnitude of decrease in HbR concentration) is greater in the social condition. Arrow A points out the significantly larger mean HbO increase in response to the social condition in the right posterior superior temporal sulcus. Differences shown for significance threshold*p*< 0.05 (Bonferroni corrected). Image reconstruction performed with data from good channels <40 mm.

**Fig. 8.**
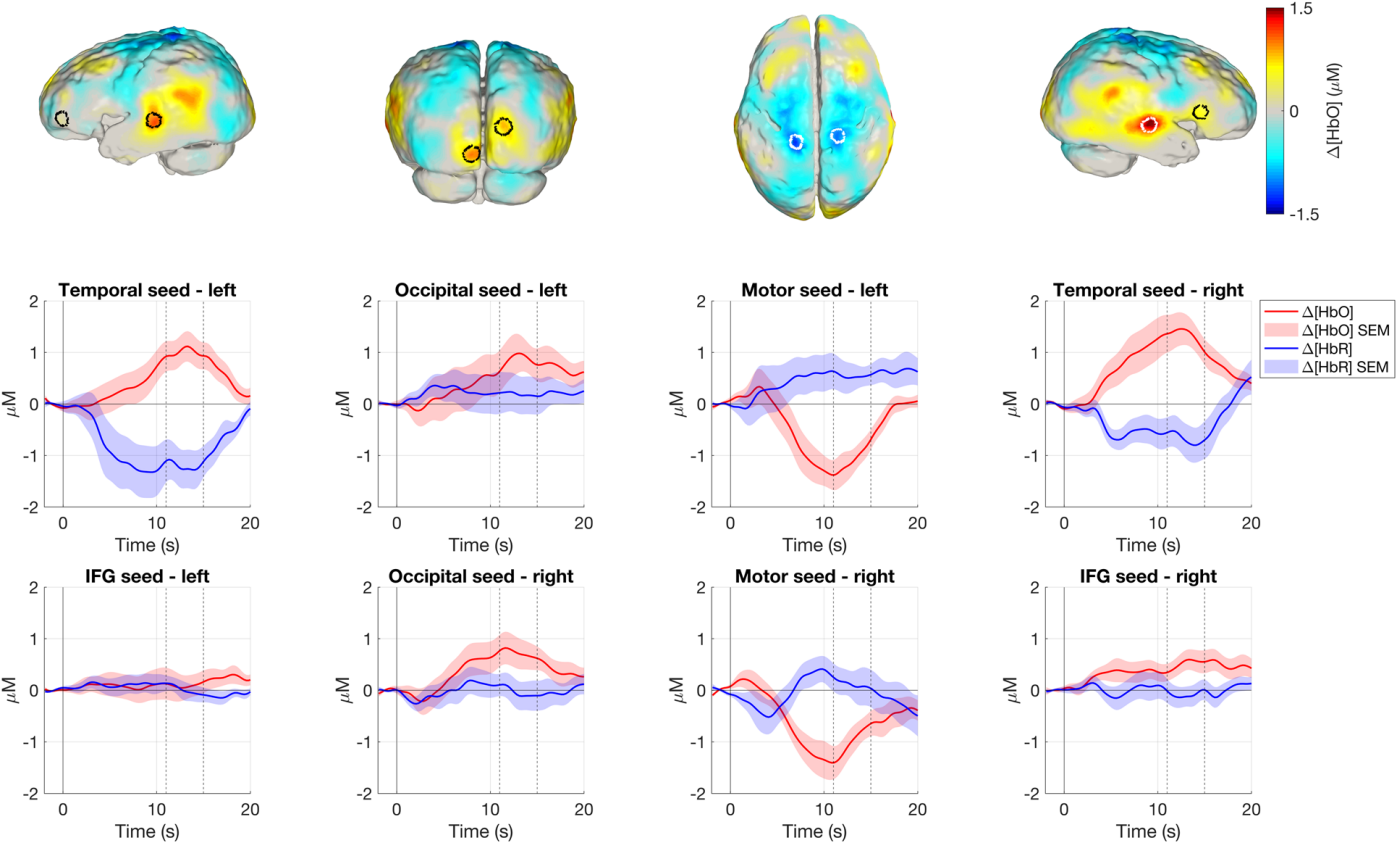
Top row: Group-level images of mean changes in HbO concentration in an 11–15 s window post-stimulus in response to the social condition, upon which the seed regions are overlayed for inferior frontal gyrus and temporal lobe (far left and far right), occipital lobe (centre left), and pre-central gyrus (centre right) overlayed on the group-level mean HbO concentration change image in response to the social condition. Middle and bottom rows: mean group-level time-courses for each seed region displaying HbO and HbR concentration changes in a 22 s period from 2 s before presentation of the social condition. Shaded area is ± standard error of the mean (SEM). IFG: inferior frontal gyrus. Image reconstruction performed with data from good channels <40 mm.

**Fig. 9.**
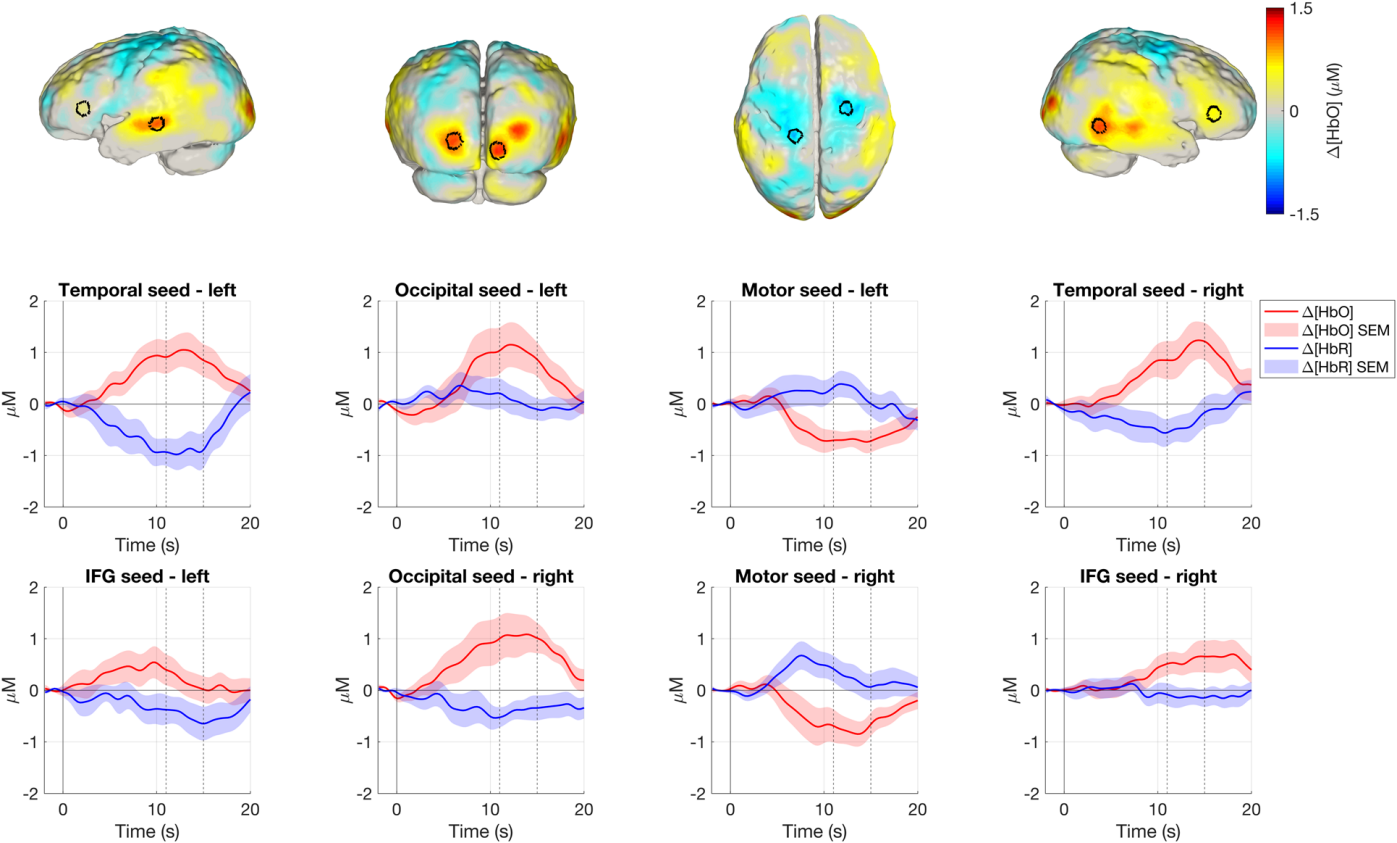
Top row: seed regions for inferior frontal gyrus and temporal lobe (far left and far right), occipital lobe (centre left), and pre-central gyrus (centre right) overlayed on the group-level mean HbO concentration change image in response to the non-social condition. Middle and bottom rows: mean group-level time-courses for each seed region displaying HbO and HbR concentration changes in a 22 s period from 2 s before presentation of the non-social condition. Shaded area is ± standard error of the mean (SEM). IFG: inferior frontal gyrus. Image reconstruction performed with data from good channels <40 mm.

InFigure 6, there is a greater area of significant response across the cortex for the non-social condition. This is further supported by the statistical comparison map inFigure 7, which shows a larger area across the cortex where haemodynamic markers of activation (i.e., significant increases in HbO and/or decreases in HbR) are greater for the non-social versus social condition. The ability to image activity across the cortex thus provides further support for the argument that there is greater spatial selectivity for the pSTS-TPJ in response to social stimuli as compared to non-social stimuli.

Taken together, our results have clearly and successfully replicated findings from several previous social fNIRS studies of the temporal cortex.

#### 4.2.2 Activation outside temporal areas

Activation is seen in the posterior occipital cortex in response to both conditions, which suggests that a visual response is occurring. In the occipital lobe, activation is seen in medial areas which extends further laterally in response to the non-social condition (seeFig. 6, arrow D). FromFigure 8, the haemodynamic response in the occipital lobe for the social condition appears to be a broader peak and of a lower magnitude compared to the temporal lobe. This difference in the response may reflect cross-cortex differences in the haemodynamic response, and is supported by the findings ofIssard and Gervain (2018), who report that canonical responses in the occipital cortex appear later than in the temporal cortex.

In response to the social condition, we see a functional response in the medial frontal gyrus which is not present in the non-social condition (seeFig. 6, arrow G). Similarly,Blasi et al. (2011), found a greater response in this region to neutral vocalisations relative to non-voice auditory stimuli (using fMRI with a cohort of sleeping infants aged 3 to 7 months).Naoi et al. (2012)found the infant medial prefrontal cortex to be voice sensitive, whileDeen et al. (2017)demonstrated a preferences to faces versus scenes in the medial frontal gyrus in awake 4- to 6-month-olds. The medial prefrontal cortex has strong connections with the hippocampus and amygdala, regions implicated in memory and emotional processing, respectively (Grossmann, 2013). This tentatively explains the involvement of the medial frontal gyrus in processing the social stimuli which contain emotional content and likely reflects experiences (e.g., hearing adults recite nursery rhymes) that the infant has already encountered.

We see more clear involvement of the inferior frontal gyrus (particularly in the right hemisphere) in response to the non-social condition. This diminished response to the social condition contrasts with previous reports of inferior frontal responses from infants in this age-range (Collins-Jones et al., 2021;Lloyd-Fox et al., 2012,^2017^). The social condition in this present work had a matched audio and visual component, whereas vocal sounds were coupled to an unrelated visual component in the studies referenced. This difference in stimulus presentation may explain the difference in inferior frontal responses, though the results in this work are not conclusive.

In posterior frontal and superior parietal regions, centred on the sensorimotor cortex, an apparent inverted response occurs, with a significant decrease in HbO concentration and a concurrent significant increase in HbR concentration. The observation is potentially due not to a genuine inverted response to the experimental conditions, but because of increased sensorimotor activation during the baseline condition. InSupplementary Figure S5, we plotted the block-averaged haemodynamic response results for the baseline condition, which shows an increase in HbO concentration and a decrease in HbR concentration in the motor seeds bilaterally following the onset of the baseline condition. Our interpretation is that this apparent increase in sensorimotor activation in the baseline period may be explained by increased movement, which subsides as the infant’s attention is drawn at the onset of the experimental conditions.

### 4.3 Limitations

The flat shape of the LUMO tile was chosen to maximise comfort of the infant. However, a limitation of this flat form is that there is a greater risk of hair blocking the optical path, which is a barrier to ensuring good contact between sources/detectors and the scalp. In the channels beyond 40 mm inFigure 4, we saw consistency in cortical coverage decrease from anterior to posterior (where more hair is located on average in this population) despite maintaining widespread consistent cortical coverage in channels <40 mm. It might well have been beneficial to have had light guides (of length a few mm) fitted to the cap underneath the tiles to improve light delivery and collection through the hair, as is common practice for adult versions of the LUMO system. This would likely have enabled us to recover a higher proportion of channels >40 mm, but may potentially make the cap more uncomfortable for infant subjects.

A limitation of the block-averaging analysis approach is that it relies on a pre-defined window to represent activation and so does not capture the temporal shape of the response, therefore not requiring the response to be associated with a canonical haemodynamic response. In addition, the Bonferroni correction was performed based on the number of nodes covered in the cortical surface mesh, which is highly conservative. Future analyses in infant HD-DOT could employ a general linear model approach to model the haemodynamic response, employing methods developed previously such as the work ofHassanpour et al. (2014),Hernandez-Martin et al. (2017),Sherafati et al. (2022), andSchroeder et al. (2023). Determining age-appropriate parameters for the canonical haemodynamic response in infants aged 5 to 7 months is critical to support the implementation of such approaches in this population.

InFigures 8and9, HbO and HbR levels appear to not fully reach baseline by 20 s post-stimulus for some of the cortical seeds, demonstrating a limitation in the paradigm design. Baseline periods would ideally be longer to allow the response to return to its pre-stimulus levels or potentially shorter stimulus periods could have been used. The competing priority here is reducing the risk of infant fussiness to maintain gaze by minimising the length of the baseline period.

Another limitation of our work is that, with the averaged MRI data used to produce the head model, we were not able to reliably map our data to an inflated cortical surface atlas to demonstrate sulcal coverage of the array and more clearly display activity in sulci.

Infant movement made it very difficult to obtain multiple, overlapping surface scans of the entire infant scalp. The most consistently high-quality scan across participants was that of the anterior of the face, and so a method (leveraging quantitative photogrammetry-derived measures) was devised, based on estimating rotations of the cap, to register optode positions to the head model. This method, however, required assumptions to be made about rotations of the cap. The difficulties encountered in this project underline the importance of fast and quantitatively validated photogrammetry methods for infant optode registration.

### 4.4 Future applications of whole-head HD-DOT

A potential application of functional neuroimaging is predicting the onset of symptoms of neurodevelopmental conditions. One example is identifying early markers of autistic spectrum condition.Lloyd-Fox et al. (2018)followed a cohort from infancy to toddlerhood. Using fNIRS, the authors report lower activation to social stimuli at 4 to 6 months was present in the pSTS-TPJ region in infants who went on to receive an autism diagnosis, demonstrating the potential predictive power of early functional neuroimaging investigations. Further, a recent fNIRS study byBlanco et al. (2023)demonstrated that infants at elevated likelihood of autism and attention-deficit hyperactivity disorder display reduced vocal selectivity in left temporal regions. To be able to apply these results predictively, larger sample sizes will be required in future research.

Research using fMRI also demonstrates that early differences in brain activity could be predictive of autism onset. For instance,Ciarrusta et al. (2019)found that neonates with a family history of autism exhibited differential activity in regions associated with social function.Ilioska et al. (2023)found that autistic adults and children exhibit alterations in functional connectivity across cortical and subcortical systems. Further,McKinnon et al. (2019)found the involvement of regions across the cortex underlies the emergence of restricted and repetitive behaviours in infants with a higher familial likelihood of autism.

These fMRI studies indicate that differences in brain activity are distributed across the infant cortex and require a much larger field-of-view than is offered by headgear used in previous infant fNIRS research. Despite advances in fMRI to enable awake infants to be scanned, movement still needs to be constrained, which limits the ability of fMRI to recreate naturalistic social contexts such as interacting with a caregiver.

Combined with its portability, ease of set-up, and high tolerance among infants, whole-head HD-DOT offers the ability to obtain cortex-wide measures across an unprecedentedly large field-of-view outside of a conventional scanner setting. Whole-head HD-DOT requires fewer resources to run and reduces potential for infant stress to improve attrition rate; this would facilitate its translation for clinical applications, making brain imaging more accessible, indicating clear potential for its widespread application in clinical diagnosis prediction. This would enable interventions to be put in place as early as possible in life to support children with neurodevelopmental conditions and their families.

## 5 Conclusion

In this study, we have conducted the first ever demonstration of whole-head HD-DOT in infants. Using a prototype HD-DOT system and custom infant headgear covering the entire infant scalp, we achieved a field-of-view mapping high-quality measures of brain activity across the entire optically-accessible cortex in infants. We have corroborated previous findings of activity in the infant social brain, finding bilateral temporal responses to audio-visual stimuli, with the largest response lateralised to the right posterior superior temporal sulcus. We have also mapped activity outside the temporal areas typically sampled in infant fNIRS research, resolving differences in the response to social and non-social stimuli in the posterior occipital cortex, inferior frontal gyrus, and medial frontal gyrus. Non-social stimuli resulted in a more widespread distribution of functional responses across the cortex. In future, the application of whole-head HD-DOT will open new opportunities to map activity in the awake infant brain in a multitude of research contexts outside conventional scanning environments to better understand the trajectory of typical and atypical neurodevelopment.

## Ethics Statement

Written, informed consent was obtained from a parent/guardian for each participant prior to their participation. Ethical approval for this study was granted by the Departmental Ethics Committee of the Department of Psychological Sciences at Birkbeck, University of London (approval reference number 2122017).

## Data and Code Availability

The data presented here were acquired under ethical approval from the Departmental Ethics Committee of the Department of Psychological Sciences at Birkbeck, University of London. Our ethical approval does not, at present, permit the public sharing of participant data. The code used in this paper for image reconstruction has been developed and released viawww.github.com/DOT-HUB.

## Author Contributions

Liam H. Collins-Jones: Conceptualisation, methodology, software, formal analysis, investigation, data curation, writing—original draft, writing—review & editing, visualisation, project administration, and funding acquisition. Louisa K. Gossé: Methodology, software, resources, investigation, and writing—review & editing. Borja Blanco: Methodology, software, resources, writing—review & editing, and formal analysis. Chiara Bulgarelli: Methodology, software, formal analysis, resources, and writing—review & editing. Maheen Siddiqui: Methodology, software, and resources. Ernesto E. Vidal-Rosas: Methodology, software. Nida Duobaitė: Methodology, software. Reuben W. Nixon-Hill: Methodology, software. Greg Smith: Methodology, software. James Skipper: Methodology, software. Tim Sargent: Methodology, software. Samuel Powell: Methodology, software. Nicholas L. Everdell: Methodology, software. Emily J.H. Jones: Resources, writing—review & editing, and project administration. Robert J. Cooper: Conceptualisation, methodology, resources, writing—review & editing, and project administration.

## Funding

Liam H. Collins-Jones was supported by the Engineering and Physical Sciences Research Council (EP/T517793/1). Louisa K. Gossé/Emily J.H. Jones was supported by awards from the Medical Research Council (MR/T003057/1) and EC Marie-Curie ETN grant 814302 (SAPIENS). Chiara Bulgarelli was supported by the Early Career Fellowship Leverhulme Trust (ECF-2021-174). Borja Blanco was supported by Medical Research Council Programme Grant MR/T003057/1 and UKRI Future Leaders fellowship (grant MR/S018425/1).

## Declaration of Competing Interest

This paper involves the application of a prototype technology developed by Gowerlabs Ltd., a company to which some of the authors are affiliated as denoted in the author list affiliations.

## Acknowledgments

We thank all parents/guardians and babies who volunteered their time to participate in this study.

## Supplementary Materials

Supplementary material for this article is available with the online version here:https://doi.org/10.1162/imag_a_00244.

## Supplementary Material

Supplementary Material
