# Supplementary material for: Whole-head high-density diffuse optical tomography to map infant audio-visual responses to social and non-social stimuli
Source: Imaging Neurosci (Camb). 2024 Sep 11;2:imag-2-00244. doi: 10.1162/imag_a_00244 (PMC12272203; doi:10.1162/imag_a_00244)
Supplement: Supplementary Material [file imag_a_00244-supp.pdf]

Supplementary material

System performance measures for each participant in the final sample.

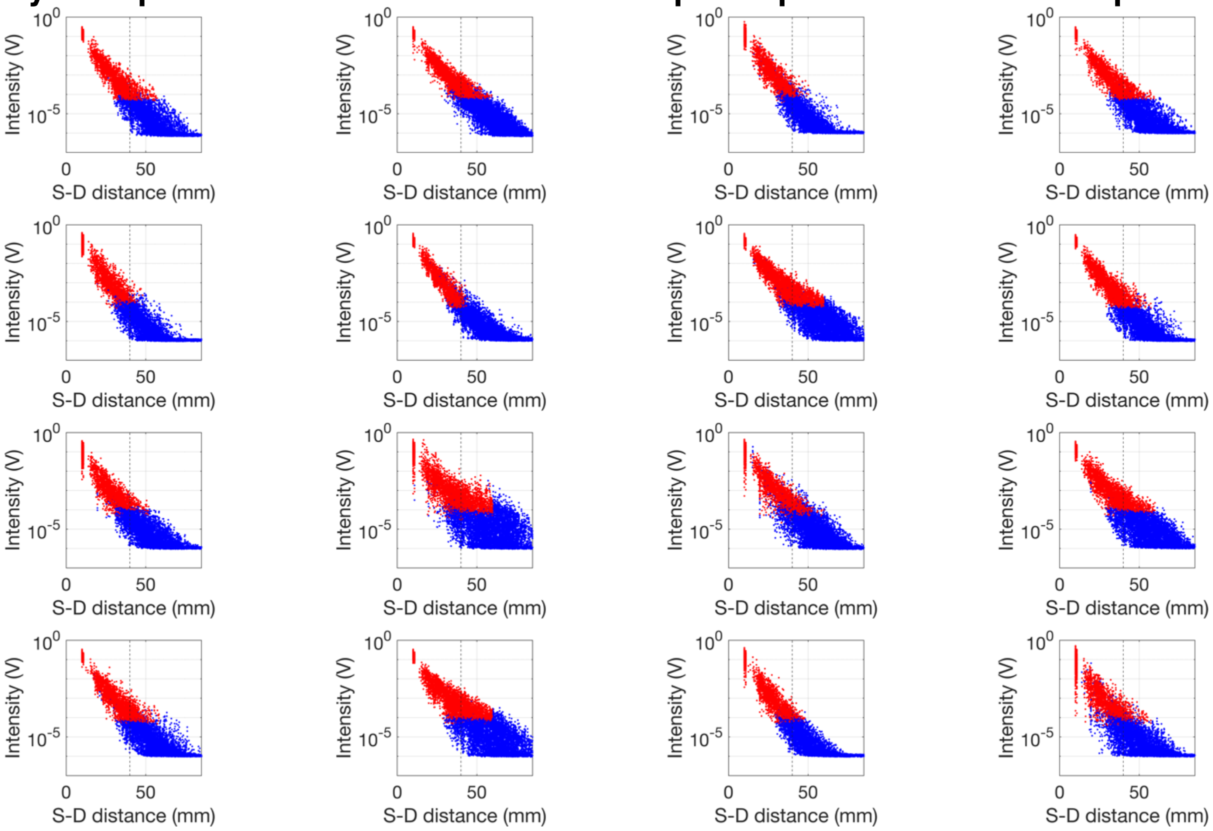

Supplementary Figure S1: Mean intensity over the time-course for each source-detector pair at each wavelength as a function of source-detector separation is plotted on a logarithmic scale for each participant (N = 16) included the final sample. In each figure, a dotted line appears at 40 mm, denoting the maximum source-detector separation used in image reconstruction.

Map of good channels across participants

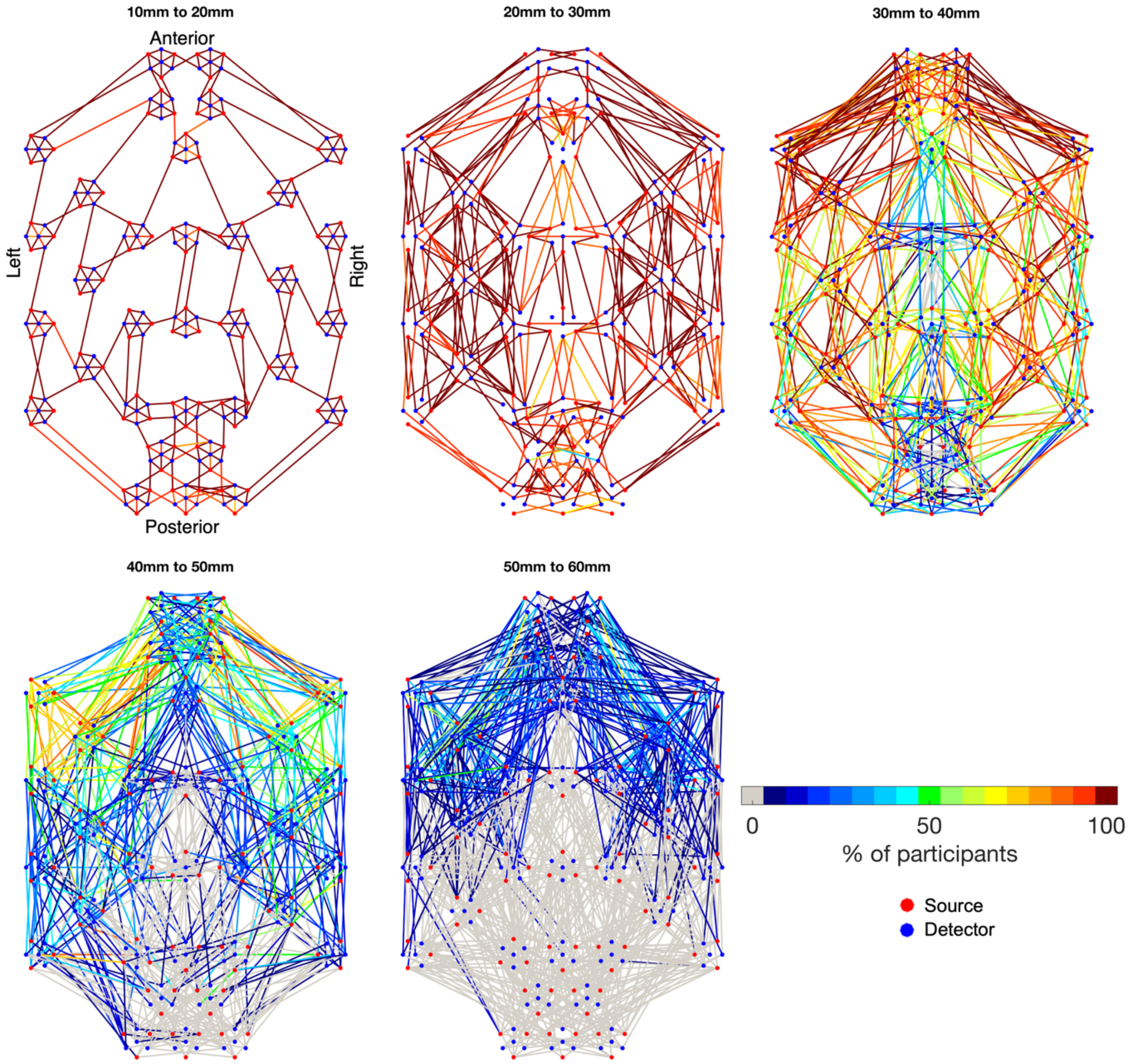

Supplementary Figure S2: Map of channels at different source-detector separation intervals, colour-coded by the frequency that the channel is classed as a good channel across participants (see colourbar). We see a high-level of consistency in good channels across participants for the 10-20 mm and 20-30 mm intervals. In the 30-40 mm intervals we see a decrease in good channels close to the midline, while above 40 mm there is a marked decrease in channel consistency across participants, with channels in anterior and lateral regions more likely to be good channels.

Haemoglobin concentration changes maps

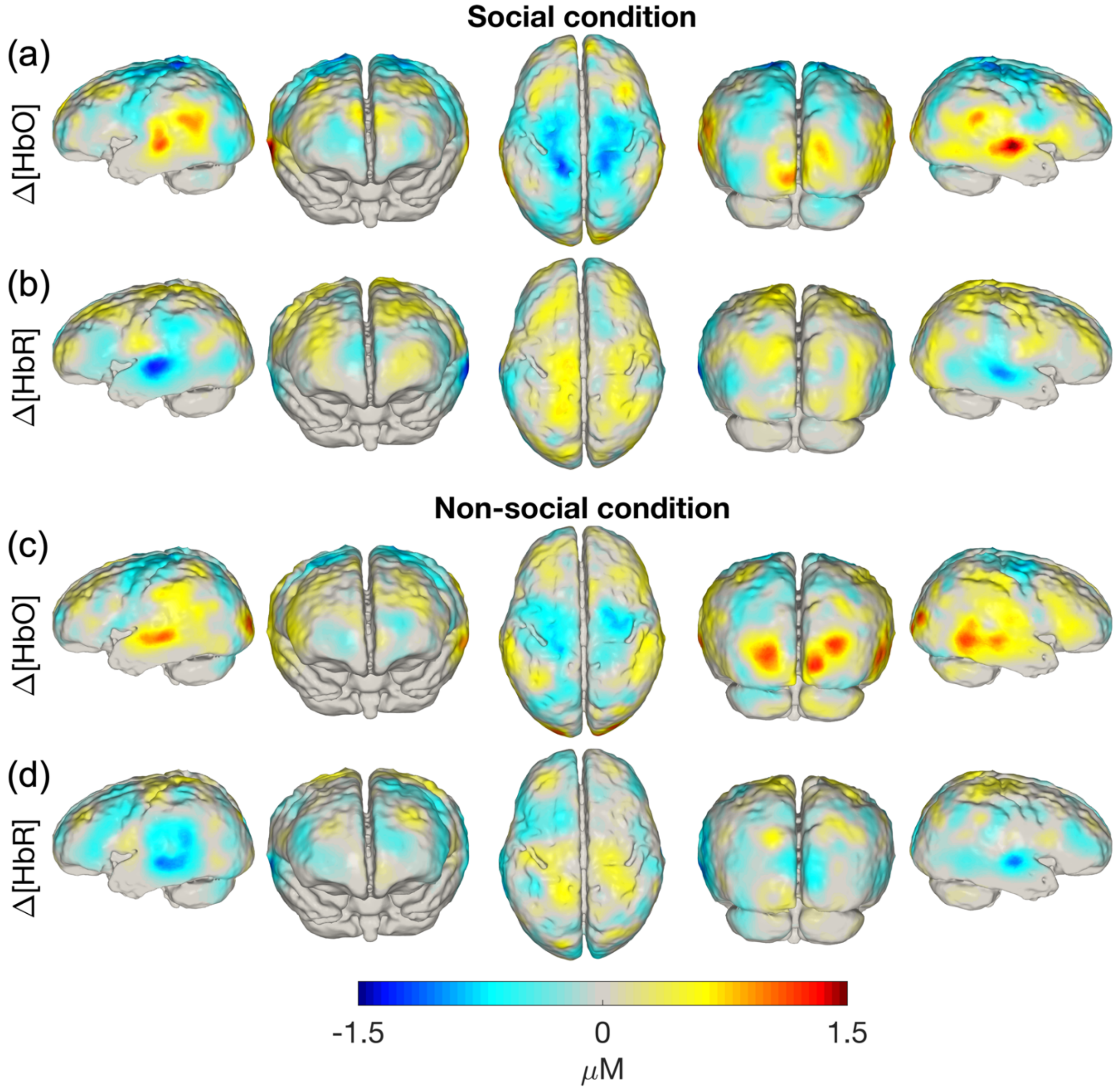

Supplementary Figure S3: Group-level images of mean changes in HbO (a & c) and HbR (b & d) concentrations in an 11-15 s window post-stimulus in response to the social (a & b) and non-social (c & d) conditions. Image reconstruction performed with data from good channels < 40 mm

T-statistic maps without annotations

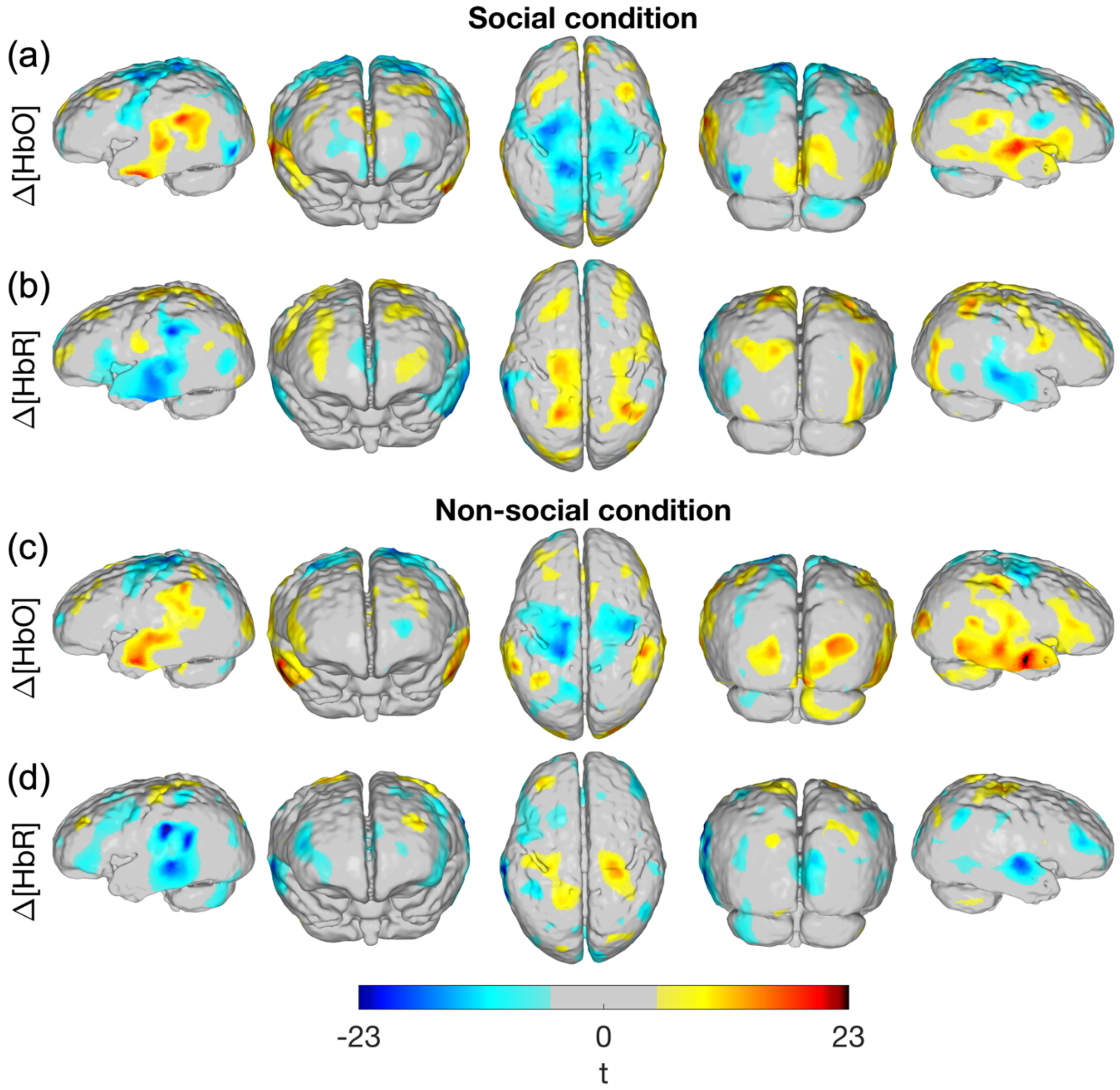

Supplementary Figure S4: t-statistic maps of the HbO (a & c) and HbR (b & d) concentration changes during an 11-15 s post-stimulus window. Results are shown for the social condition (a & b) and the non-social condition (c & d). Changes shown for significance threshold $p < 0.05$ (Bonferroni corrected). Image reconstruction performed with data from good channels < 40 mm. Figure 6 displays the same image but with annotating arrows and circles to highlight key areas of activity.

1.1 Block-averaged concentration change time-courses for the baseline condition

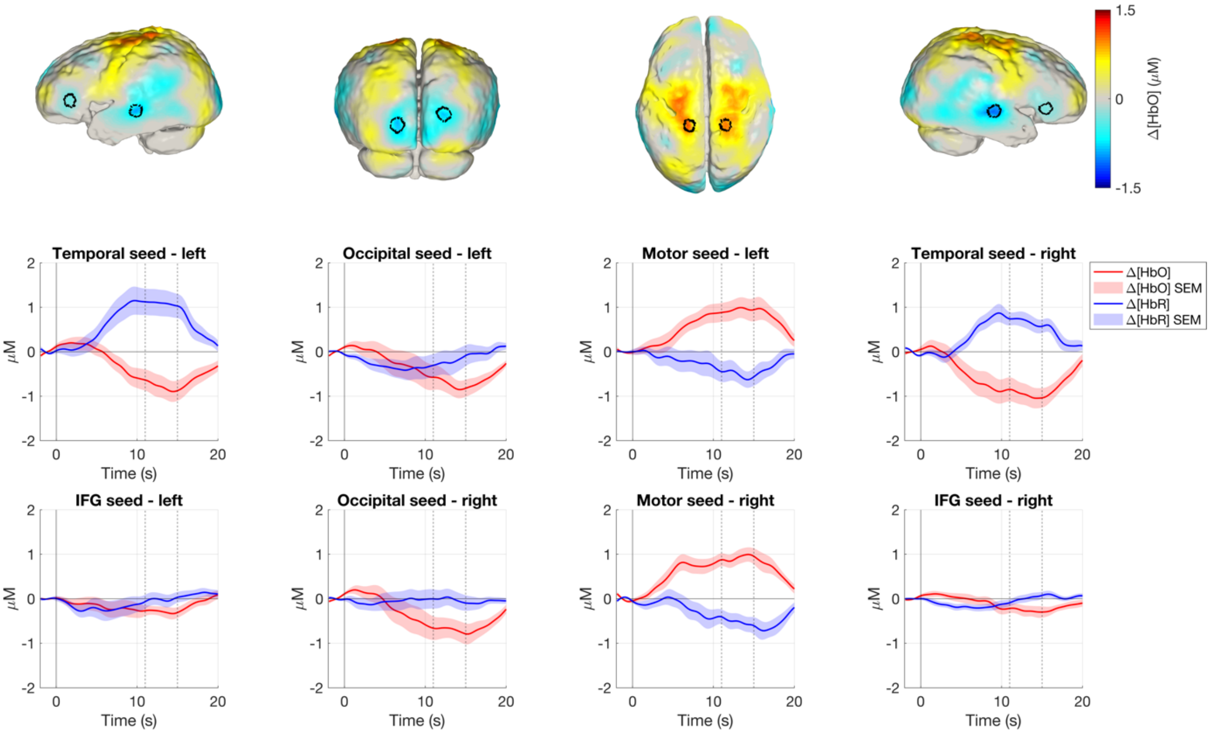

Supplementary Figure S5: Top row: seed regions for inferior frontal gyrus & temporal lobe (far left and far right), occipital lobe (centre left) and pre-central gyrus (centre right) overlayed on the group-level mean HbO concentration change image, using the onset of the baseline condition (at time = 0) as the event marker. Middle and bottom rows: mean group-level time-courses for each seed region displaying HbO and HbR concentration changes in a 22 s period from 2 s before the onset of the baseline condition. Shaded area is ± standard error of the mean (SEM). IFG: inferior frontal gyrus. Image reconstruction performed with data from good channels < 40 mm.
